# Identifying postoperative complications after inguinal hernia repair with a smartphone application: a comparative cohort study

**DOI:** 10.1007/s10029-024-03019-7

**Published:** 2024-03-20

**Authors:** L. van Hout, M. J. R. Harker, P. W. H. E. Vriens, W. J. V. Bökkerink

**Affiliations:** 1 Department of Medical and Clinical Psychology, Tilburg School of Social and Behavioral Sciences, Tilburg University, Tilburg, The Netherlands; 2 Department of Surgery, Hernia Centre Brabant, Elisabeth-TweeSteden Hospital (ETZ), Tilburg, The Netherlands; 3 Department of Surgery, Radboud University Medical Centre, Radboud University, Nijmegen, The Netherlands

**Keywords:** mHealth, Smartphone application, Inguinal hernia, PROM, Validity

## Abstract

**Purpose:**

The Q1.6 Inguinal Hernia application continuously measures patient-reported outcomes (PROs) by sampling experiences through brief, digital and condition-specific questions, utilising micro-moments. This can overcome the limitations of current paper questionnaires and give real-time insight into patient recovery. This exploratory study compares data from the application with retrospective data from electronic medical records (EMRs) to provide information on its accuracy in detecting postoperative complications after inguinal hernia repair.

**Methods:**

Patients were asked to use the application in addition to their usual care. The application employs *twitch crowdsourcing* to gather PROs. Questions from validated and frequently used questionnaires were integrated. A retrospective assessment of EMRs was combined with an additional telephone interview. The primary endpoints were the sensitivity and specificity of the application in detecting chronic postoperative inguinal pain, recurrence and surgical-site infection (SSI).

**Results:**

A total of 215 patients were analysed. The sensitivity and specificity for detecting chronic postoperative inguinal pain were 100% (95% CI [47.8%, 100%]) and 93.7% (95% CI [88.3%, 97.1%]), respectively. For recurrence, the sensitivity was 77.8% (95% CI [40.0%, 97.2%]), and the specificity was 81.3% (95% CI [75.0%, 86.5%]). For SSI, the sensitivity and specificity were 75.0% (95% CI [19.4%, 99.4%]) and 89.8% (95% CI [84.8%, 93.6%]), respectively.

**Conclusion:**

This study demonstrates satisfactory measurement capabilities of the Q1.6 Inguinal Hernia application for identifying postoperative complications following inguinal hernia repair. However, certain aspects require further improvement, such as addressing error-prone questions, enhancing long-term compliance, and validating (pain) measurements through prospective control data.

**Trail registration number:**

NL7813 (Dutch Trial Registry), 19 May 2019.

## Introduction

Inguinal hernia surgery outcomes are commonly assessed through a single doctor’s visit or a telephone call, often underexposing patient-reported outcomes (PROs) [1]. However, important outcomes like chronic postoperative inguinal pain (CPIP) and recurrence require long-term follow-up [2]. The currently available measuring instruments, typically involving lengthy questionnaires administered at fixed intervals, are rarely used in routine clinical practise. Moreover, they have the potential to introduce biases and lead to suboptimal compliance or timing [3]. To address these challenges, the Q1.6 Inguinal Hernia application was developed. This mobile device application continuously measures PROs by sampling experiences through brief, digital and condition-specific questions, utilising micro-moments [3, 4].

The technical, practical, legal and ethical background of this application has been previously described *(i.e. technical feasibility)* [3]*.* Furthermore, the first experiences regarding its feasibility and practicability in daily practise have been documented *(i.e. clinical feasibility)* [4]*.* The application effectively collected a significant amount of data, enabling repeated measurements and personalised outcome sets. Patients were able to report preoperative and postoperative clinical outcomes, and there was high patient satisfaction. In addition, the application incorporates a notification system to detect suspected complications [4].

Remote patient monitoring must be assessed for validity, safety and cost-effectiveness before it can be considered a viable alternative to conventional post-inguinal hernia repair follow-up. This study represents an initial step in this validation process: in an exploratory study, prospectively collected data (PROs) using the Q1.6 Inguinal Hernia application are compared with retrospectively obtained clinical data from electronic medical records (EMRs). To enhance the reliability of the comparison, the reference data from the EMRs were verified through follow-up telephone interviews. The study aimed to provide initial insight into the accuracy (*i.e. clinical validation*) of the Q1.6 application in detecting postoperative complications (*i.e. clinical safety*).

## Methods

### The Q1.6 Inguinal Hernia application

**Fig. 1:**
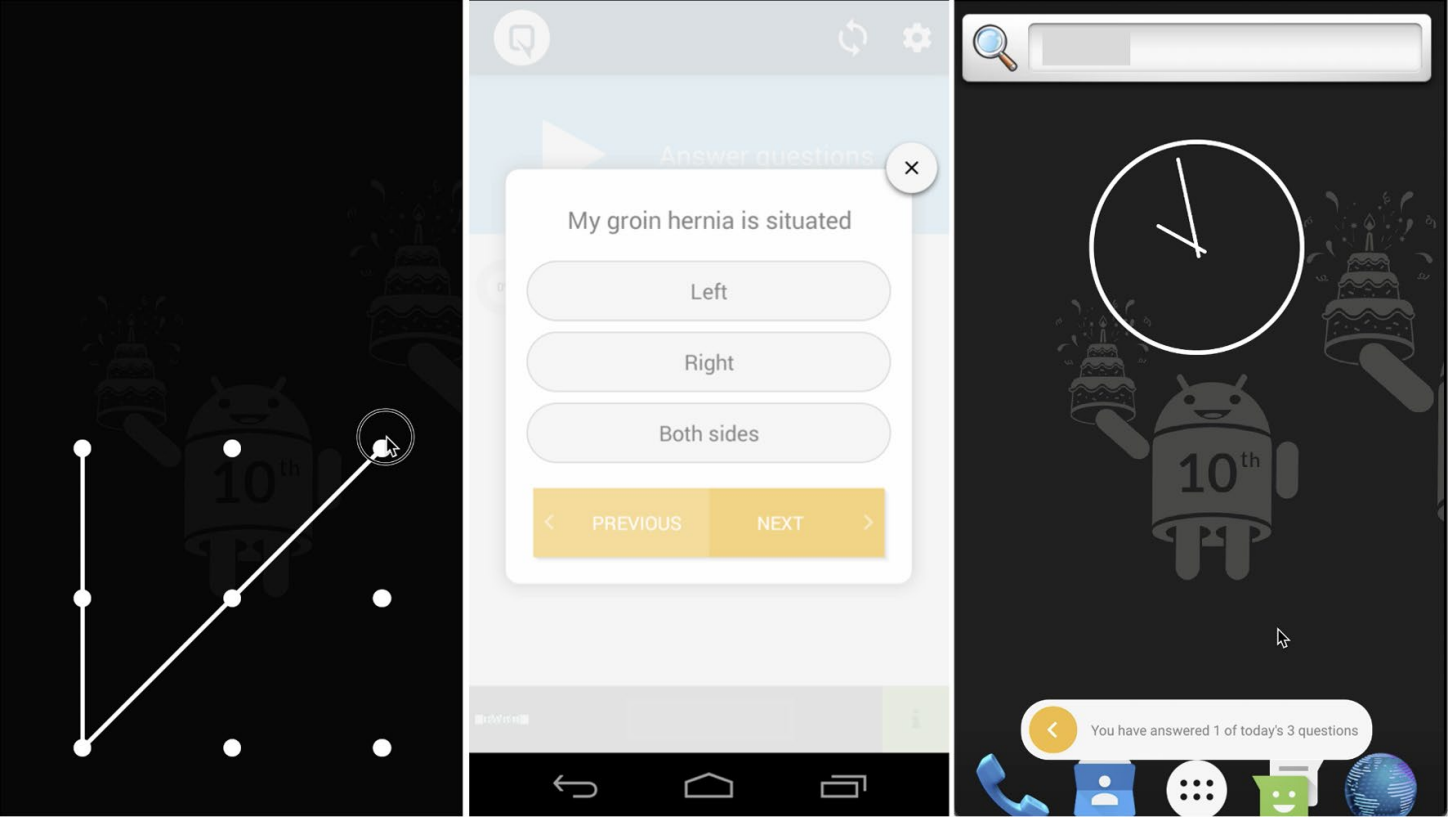
Following the unlocking of a smartphone’s screen (micro-moment), a concise query is presented

The digital Q1.6 platform employs *twitch crowdsourcing* to collect real-time data from patients [5]. Within the short interval or micro-moment after unlocking a smartphone or tablet, a brief and readily answerable question is presented (Fig. 1). When responded to within 1.6 s, it does not impact accuracy or delay in working memory compared to a standard unlock gesture [6]. As a result, it is not perceived as annoying or intrusive and can be done multiple times a day, week, or month. Disease-specific questions derived from validated and frequently used questionnaires were integrated [7]. The adaptive question engine incorporates an algorithm that adjusts questions and frequencies based on the provided answers, yielding unique and individualised outcome sets. Outcomes are continuously monitored and available in real time on a web-based dashboard accessible to treating physicians. In addition, a notification system is available when answers may indicate the occurrence of a complication. In such instances, an alert is displayed on the dashboard, and an email notification is sent to the treating physician.

### Study design and participants

This exploratory study aims to validate prospectively obtained data from the Q1.6 Inguinal Hernia application by comparing it with retrospective data from the corresponding EMRs, verified with an additional telephone interview. The patient cohort was assembled prospectively between September 2016 and March 2018 at the Elisabeth-TweeSteden Hospital (Tilburg, the Netherlands). Patients scheduled for elective inguinal hernia repair were invited to use the application as an additional monitoring tool alongside their usual care. Usual care included a preoperative surgical consultation, a 2-week standard postoperative follow-up visit, and subsequent visits or telephone calls upon indication. Patients were excluded from participation if they were not adults, had insufficient Dutch language proficiency, cognitive limitations, or were lacking a smartphone or tablet with Apple’s iOS or Google’s Android operating systems. Participation was voluntary and uncompensated. The application is free of charge, and it does not require any in-app purchases. After providing written informed consent, patients received a unique activation code to install the application. During the 1-year follow-up, patients used the application without any obligation or digital or oral reminders.

### Data collection

After the conclusion of the 1-year follow-up period of the pilot study, Q1.6 provided a pseudonymized database, which was accessible only to affiliated researchers with a decryption key. Between June 2020 and August 2020, the corresponding EMRs of participating patients were retrospectively assessed to collect clinical data, including demographics, preoperative, (peri)operative, and postoperative information, as well as any inguinal hernia surgery-related complications. Furthermore, in August and September 2020, patients were contacted by phone and asked five questions (Table 1). Questions 1 to 4 concern a validated telephone tool, the PINQ-PHONE questionnaire, designed for recurrent inguinal hernia screening with an overall sensitivity and specificity of 1.00 and 0.85, respectively [8]. Particularly, questions 3 and 4 (enhanced PINQ-PHONE) demonstrated the best positive predictive value for the presence of a recurrence [9].

**Table 1:** Questions asked during the telephone interview

1	Do you have any symptoms in your operated groin?
2	Have you noticed anything at your operated groin?
3	Have you noticed something at the operated groin when coughing, sneezing or squeezing?
4	Could you please stand up and put your other hand flat in your operated groin. Now please put the phone down, put this hand to your mouth, and blow. Do you feel something in your operated groin?
5	Have you been operated on at a different hospital for a recurrent inguinal hernia?

### Endpoints

The primary outcomes of this study were the sensitivity and specificity of the Q1.6 Inguinal Hernia application in detecting three postoperative complications within the first year after surgery: chronic postoperative inguinal pain (CPIP), recurrent inguinal hernia and surgical-site infection (SSI). CPIP was defined as clinically relevant pain persisting for more than 3 months postoperatively [2, 10]. For the clinical data (EMRs), this encompassed all relevant pain reported during outpatient department interviews or when patients sought treatment for CPIP. During the additional telephone interview, patients were asked about any CPIP treatment received elsewhere or relevant complaints experienced during the first year after surgery. For data obtained from the Q1.6 application, clinically relevant pain was defined as pain at the surgical site with a numeric rating scale (NRS) score of ≥ 4 (i.e. moderate to severe pain) after the third postoperative month [10]. Recurrent inguinal hernia was defined as any recurrence diagnosed by a physician on physical examination within 1 year of follow-up. During the telephone interview, patients were asked recurrence-related questions (PINQ-PHONE, Table 1), and they were also questioned about any occurrences of recurrence more than 1 year postoperatively or any treatments received for recurrence elsewhere. Regarding the Q1.6 application, any reported presence of a new bulge in the operated groin during follow-up was considered a suspected recurrence. However, these suspected cases were not clinically verified. To assess SSI, the CDC definition was applied [11], including a) purulent drainage from the incision; b) isolation of organisms from local tissue or aspirated fluids; and c) deliberate reopening of the wound, combined with at least one sign of localised pain or tenderness, localised swelling, erythema or heat within 30 days after the initial procedure. The presence of these criteria was checked in the EMRs, and culture results were examined when available. In the Q1.6 database, patients were suspected of having an SSI if they answered “Yes” to the questions “Do you have a fever?” and/or “Is there purulent leakage from the wound?” or “Yes” twice in a row to the questions “Is the skin surrounding the wound red?” and/or “Does the wound feel warm?”. These questions were posed when a patient-reported inadequate wound healing (“Does the wound heal well?”) during the first 2 postoperative weeks. Since the Q1.6 application used a twitch crowdsourcing concept with repeated questions, individual patients who met the definition for any primary outcome more than once during follow-up were considered only once for each complication. Cases with missing data for a specific complication were excluded from the diagnostic accuracy calculations.

Secondary outcomes included baseline characteristics (age, gender, body mass index, and ASA classification), preoperative data, and operative details (inguinal hernia repair technique and EHS Groin Hernia Classification [12]. These were extracted from EMRs because the Q1.6 application does not store clinical and patient-identifiable information.

### Data security and storage

Except for the operation date, the application did not request or store personal data. A unique activation code was needed during installation. This unique code encrypts patient information (i.e. pseudonymization) and is used to display cases on the password-protected web-based dashboard. Data collected with the application were stored on the servers of the Q1.6 platform (Amazon Web Services, Dublin, Ireland) for a maximum of 2 years. This platform complies with the international legislation and certification obligations that apply to the storage of medical data (e.g. GDPR, CE marking, ISO 27001 and ISO 13485 certification). Q1.6 is only involved as a data processor, as legally warranted in a Data Processing Agreement, and has no access to or influence over the data itself. Data retrieved from EMRs were stored in a digital database that has been secured and stored in accordance with applicable in-hospital regulations.

### Statistical analysis and reporting

Sensitivity and specificity were calculated for postoperative complications (CPIP, recurrent inguinal hernia and SSI) along with their corresponding 95% confidence intervals. Continuous variables were presented as mean ± standard deviation (SD), whilst qualitative or categorical variables were expressed as frequencies and percentages. NRS scores were reported as mean ± standard deviation (SD). Descriptive statistical analysis was conducted using IBM SPSS Statistics for Windows, version 25.0.0.1 (IBM Corp., Armonk, NY, United States). The report adhered to the Strengthening The Reporting of Observational studies in Epidemiology (STROBE) recommendations for cohort studies [13].

## Results

A total of 229 patients installed the first (pilot) version of the Q1.6 Inguinal Hernia App to test its clinical feasibility [4]. All participants provided informed consent before installation. From this cohort, the EMRs of 215 patients were assessed, with 14 EMRs unable to be traced due to human errors in the encryption list. A follow-up telephone interview (Table 1) was conducted with 184 (85.6%) of the 215 analysed patients. Baseline characteristics and operative data obtained from EMRs are presented in Table 2. Most inguinal hernias (78.1%) were repaired with the TransInguinal PrePeritoneal (TIPP) technique, which is the standard procedure for unilateral symptomatic inguinal hernias in the Elisabeth-TweeSteden Hospital (Tilburg, The Netherlands).

**Table 2:** Baseline patient characteristics and operative data from EMRs (*n* = 215)

			*n*	*%*
Gender				
	Male		202	94.0
	Female		13	6.0
Age (mean ± SD)			55.40 ± 13.55	
ASA classification				
	I		83	38.6
	II		46	21.4
	III		13	6.0
	IV		0	0.0
	Missing		73	34.0
BMI				
	Mean ± SD		25.21 ± 3.41	
	Missing		81	37.7
Operation type				
	TEP		14	6.5
	TREPP		28	13.0
	TIPP		168	78.1
	Lichtenstein		5	2.3
EHS classification				
	Lateral		43	20.0
	1		5	2.3
	2		24	11.2
	3		14	6.5
	Medial		41	19.1
	1		3	1.4
	2		9	4.2
	3		29	13.5
	Combined (pantaloon)		5	2.3
	Missing		126	58.6

*n* number of patients, *SD* standard deviation, *ASA* American Society of Anaesthesiologists, *BMI* body mass index, *TEP* Totally Extra-Peritoneal technique, *TREPP* Trans Rectus-sheath Extra-Peritoneal Procedure, *TIPP* TransInguinal PrePeritoneal technique, *EHS* European Hernia Society Inguinal Hernia Classification

### Preoperative data

To assess the agreement between PROs obtained from the application and data documented during patient interviews at surgical consultations (recorded in EMRs), certain preoperative variables were compared and are presented in Table 3.

**Table 3:** Registration of preoperative characteristics, EMR versus Q1.6 app. *n* = 215

		*n*	*n*	*n*
Hernia side		EMR—Left	EMR—Right	EMR—Bilateral
	App—Left	**63**	*3*	*1*
	App—Right	*2*	**101**	*1*
	App—Bilateral	*1*	*2*	**15**
	App—Missing	8	16	2
Preoperative symptoms		EMR—Symptomatic	EMR—Asymptomatic	EMR—Missing
	App—Symptomatic	**130**	*2*	18
	App—Asymptomatic	*27*	**9**	2
	App—Missing	21	2	4
Self-noted bulge		EMR—Yes	EMR—No	EMR—Missing
	App—Yes	**124**	*1*	41
	App—No	*5*	**10**	7
	App—Missing	18	0	9
Paid employment		EMR—Yes	EMR—No	EMR—Missing
	App—Yes	**26**	*2*	96
	App—No	*1*	**3**	57
	App—Missing	6	1	23
NRS in rest		EMR—Present	EMR—Missing	
	App—Present	0	187	
	App—Missing	0	28	
NRS during activity		EMR—Present	EMR—Missing	
	App—Present	0	187	
	App—Missing	0	28	

The agreement between the Q1.6 application and the EMRs is highlighted in **bold**, whilst the differences are highlighted in *italics*

*n* number of patients

*EMR* electronic medical record

*NRS* numeric rating scale

In 26 of 215 (12.1%) patients, the hernia side was not registered in the application. In the first version of the application (from which the current data are extracted), patients were not obligated to answer questions and were able to skip questions. From the remaining 189 patients, 179 (94.7%) registered the correct hernia side when EMR data are taken as reference. Information about preoperative complaints was available for 168 (78.1%) patients, and in 139 (82.7%) of these cases, a resemblance was seen between data from the EMRs and collected PROs in the application. The presence of a self-noted bulge was described in the EMRs of 140 (65.1%) patients, of whom 134 (95.7%) provided similar answers in the application. Table 3 also shows that the application can be of added value in obtaining information that was not recorded in EMRs by default, namely: employment status (86.0% vs. 18.1%) and NRS scores at rest and during activity (both 87.0% vs. 0.0%). The mean NRS (± SD) at rest was 1.54 (± 1.89) and 3.89 (± 2.85) during physical activity.

### Postoperative data

Data from EMRs (*n* = 215) combined with data from the additional telephone follow-up (*n* = 184) were used as a reference for the primary endpoints (CPIP, recurrence, and SSI). Within this cohort, 21 patients (9.8%) had a complicated course in the first postoperative year; 5 patients (2.3%) suffered CPIP, but in 2 of them, this was due to a recurrence, which was treated; a total of 9 patients (4.2%) experienced a recurrence; 4 (1.9%) had an SSI; and 5 (2.3%) had other complications requiring additional treatment, such as hematoma or postoperative bleeding. Meanwhile, responses to questions in the Q1.6 application indicated 81 (37.7%) possible or patient-reported complications: 14 patients (6.5%) experienced CPIP; 43 (20.0%) reported a potential recurrence; and 24 (11.2%) reported signs suggestive of SSI. Table 4 provides cross-tabulations for the primary outcomes. Based on these data, a preliminary sensitivity and specificity were calculated for the chance of detection or exclusion of a complication by the Q1.6 application. These are shown in Table 4 with 95% confidence intervals.

**Table 4:** Cross-tabulations for postoperative complications during the first postoperative year, *n* = 215

		Reference—Positive	Reference—Negative	Sensitivity [95% CI]	Specificity [95% CI]
CPIP				100% [47.8%, 100%]	93.7% [88.3%, 97.1%]
	App—Yes	**5**^a^	*9*^b^		
	App—No	*0*	**133**		
	App—Missing	0	68		
Recurrent hernia				77.8% [40.0%, 97.2%]	81.3% [75.0%, 86.5%]
	App—Potential +	**7**	*36*^b^		
	App—Potential −	*2*^b^	**156**		
	App—Missing	0	14		
SSI				75.0% [19.4%, 99.4%]	89.8% [84.8%, 93.6%]
	App—Potential +	**3**	*21*^b^		
	App—Potential −	*1*^b^	**184**		
	App—Missing	0	6		

The agreement between the Q1.6 application and the EMRs is highlighted in **bold**, whilst the differences are highlighted in *italics*

*n* number of patients, *Reference* data from EMR combined with additional telephone interview, *CPIP* chronic postoperative inguinal pain, *SSI* surgical-site infection, *CI* confidence interval

^a^In two of the five patients, CPIP was caused by a recurrence

^b^Details regarding these mismatches are described in the text

To better understand the disparities observed between the two measurement methods, a comprehensive re-examination of the EMRs was conducted to explore potential explanations. The 14 patients who reported CPIP in the application had an average follow-up duration with the application of 263.6 days, whereas they had an average of 3.9 postoperative outpatient visits spanning 85.7 days. Notably, the EMRs of nine patients did not explicitly document the presence of CPIP. However, four of these patients had three or more (3–11) outpatient visits and additional examinations due to groin complaints, and two patients disclosed the occurrence of CPIP during the additional phone interview, with one of them also receiving treatment for a recurrence after a 3-year interval. Conversely, no plausible explanation was identified in the EMRs of the remaining patients. During the first year following surgery, nine recurrences were diagnosed and treated. Two recurrences out of nine went unnoticed by the application, whilst the application was still being used by these patients at the time of diagnosis. In addition, these patients did not report any pain symptoms in the application. During reassessment of the 36 patients with a potential recurrence according to the Q1.6 application, most records indicated occurrences such as “swelling in the operated groin due to hematoma” or “thickened scar tissue”. Furthermore, five recurrences were diagnosed after follow-up with the application ended, with an average time to diagnosis of 590.2 days. In addition, during the telephone interview, three more recurrences were suspected based on the PINQ-PHONE method [8, 9], although lacking clinical verification. The Q1.6 application failed to identify one out of the four SSIs. This particular patient did not utilise the application for more than 5 days following the surgery, whilst the SSI was diagnosed on the twelfth postoperative day. In the EMRs of the majority of the 21 patients whose application responses suggested the possibility of an SSI, the described signs included “swollen wound without infection,” “hematoma,” and “serous exudate from the wound.” However, these cases were not classified and treated as SSIs.

## Discussion

The Q1.6 Inguinal Hernia application was developed to continuously measure and sample experiences or PROs after inguinal hernia repair, utilising micro-moments to present digital, concise and condition-specific questions. The primary aim of this study was to provide an initial insight into the accuracy of the application, specifically its ability to detect the following postoperative complications: chronic postoperative inguinal pain (CPIP), recurrence and SSI.

The Q1.6 application demonstrated a high capability to identify complications in the majority of cases. The sensitivity and specificity values for detecting chronic postoperative inguinal pain were 100% (95% CI [47.8%, 100%]) and 93.7% (95% CI [88.3%, 97.1%]), respectively. In terms of recurrence detection, the sensitivity was 77.8% (95% CI [40.0%, 97.2%]), and the specificity was 81.3% (95% CI [75.0%, 86.5%]). For SSIs, the sensitivity and specificity were 75.0% (95% CI [19.4%, 99.4%]) and 89.8% (95% CI [84.8%, 93.6%]), respectively.

Regarding CPIP, nine inconsistencies between the application and EMRs were identified. Explanations could be: a) insufficient documentation in EMRs: most of these nine patients exhibited an atypical course with persisting groin complaints after surgery, suggesting potential CPIP involvement, although not explicitly documented in EMRs. b) Disparities in follow-up duration: on average, the application was utilised for a period of 170 days postoperatively, in contrast to an average of 1.52 (ranging from 0 to 11) follow-up visits. Furthermore, 149 patients (69.3%) underwent a solitary follow-up visit 2 weeks after surgery. Therefore, CPIP could have been missed due to differences in follow-up duration. c) Intensity of complaints: it is conceivable that the application detected mild CPIP complaints that patients did not consider significant enough to seek medical attention. The reason for the application missing two out of nine recurrences in the first postoperative year remains unclear. However, the missed SSI can be attributed to differences in follow-up duration.

Notably, a considerable number of patients reported a suspected recurrence (*n* = 36) or suspected SSI (*n* = 21) through the application, yet these claims were never confirmed by healthcare providers. This indicates that questions can be enhanced, and modifying the question’s prompt could potentially yield beneficial outcomes. One might question the necessity of reducing false-positive responses to zero, as the ideal scenario entails detecting all clinically relevant complications without any omissions.

The baseline characteristics revealed a standard inguinal hernia population. Secondary outcomes comprised preoperative variables selected to evaluate the application’s accuracy from an alternative perspective. Overall, there was adequate agreement between the Q1.6 application responses and data obtained from EMRs, including hernia side, preoperative complaints, and self-reported bulge. However, one could raise questions about the lack of full agreement on certain parameters. For instance, regarding the hernia side and the presence of complaints, there should not be any difference in the reporting observer, whether it is the patient themselves or a doctor. However, simple explanations may account for the inconsistencies: a) human error in data collection; b) human error in answering questions; c) variations in the definition of symptoms like ‘pain’ or ‘complaints’; d) errors in sidedness (mirror view or ‘self’ view); e) cosmetic concerns. Whilst some of these explanations involve true errors, for subjective causes like varying definitions, the patient’s perspective could be considered a reference and given precedence, especially as PROs gain increasing importance in research and healthcare. Furthermore, the application captured outcomes not routinely documented during surgical consultations, such as employment status and preoperative pain scores (NRS). It also collected valuable patient-reported postoperative outcomes, including return to work, sports participation, complaint resolution, additional doctor visits and patient satisfaction [4]. In current usual care, finding these data within EMRs is exceedingly challenging, and as such, they are beyond the scope of this report.

Reviewing the literature to compare our results with similar studies revealed the existence of many mobile device applications but hardly any qualitative scientific reports evaluating them. This is remarkable given the upcoming popularity of remote monitoring, particularly since the COVID-19 pandemic. Also in the Netherlands, the use of medical and mobile device applications is now widespread [14–17].

Some studies that could be found showed interesting results. Faessen et al. [18] examined the use of an eHealth (electronic health) application for monitoring postoperative progress after inguinal hernia surgery. Patients were administered a digital survey nine times during the initial 14-day postoperative period, followed by standard follow-up. An algorithm alerted healthcare providers to deviations from normal recovery. Measured in 60 compliant patients (49%), the application demonstrated a sensitivity of 77.4% and a specificity of 57.1% for detecting abnormal pain levels, wound infection or swelling (hematoma or recurrence). Patients expressed high satisfaction. Meuzelaar et al. [19] utilised an application to educate patients about inguinal hernia treatment and recovery, offering chronologically released information and a built-in database. The app was well-received by patients, who found it user-friendly and valuable as a complementary treatment resource. Multiple preoperative and postoperative paper and digital PROM questionnaires were used. Despite low patient adherence (47%), the application demonstrated excellent reliability, convergent validity and test–retest reliability, measured in 59–77 patients. However, limited comparisons were possible due to low compliance. These conclusions align with the findings of the Q1.6 Inguinal Hernia project. However, it is important to note that direct comparisons between the studies are challenging due to variations in technology and application usage.

Given the limited availability of comparable projects, other mHealth studies examining similar outcome measures were identified. First, when considering the outcomes of pain and/or functional recovery: several studies demonstrate the feasibility and comparability of digital pain measurement tools, such as the NRS and visual analogue scale (VAS), compared to validated paper versions [20, 21]. In addition, more generally, some studies report that PRO measurement with smartphone applications enhances postoperative recovery and reduces postoperative symptoms compared to standard care [21, 22]. Overall, more studies report that measuring pain after surgical treatment with a mobile device application is suitable and safe [23, 24]. Nevertheless, it should be noted that larger (randomised controlled) trials are lacking to date. The Q1.6 application utilises digital NRS scores to measure pain, which has demonstrated feasibility and reliability in relevant studies [20]. However, validation against prospectively collected clinical pain scores is necessary to assess validity and reliability.

Second, the use of mHealth for detecting SSIs has been studied before [25, 26]. Ng et al. [26] reported in their systematic review that mobile device applications, along with clinical photos, can effectively detect SSIs with diagnostic accuracy ranging from 69.5% to 100%. Clinical photos taken by patients seem essential. Gunter et al. [27] utilised an application allowing patients to upload wound photos and complete daily SSI-related surveys to identify SSIs after vascular surgery, achieving a 90.2% submission rate. Their protocol detected 87.5% of the SSIs within 24 h. Scheper demonstrated an 80% agreement between patient-reported and physician-reported outcomes on wound problems after arthroplasty using a question-only application [28]. Recognising a SSI after inguinal hernia repair proves challenging for participating patients. The comprehensive CDC definition leads to potential confusion due to multiple questions about wound healing [11]. Incorporating options for uploading clinical photos in future versions of the Q1.6 application may improve the precise and timely identification of SSIs. High sensitivity is crucial to ensure that no SSI goes unnoticed.

Third, the detection of recurrence after inguinal hernia repair through mobile device applications is unexplored. Clinical examination is the current gold standard, but evidence suggests that recurrence can be remotely detected using the (enhanced) PINQ-PHONE questionnaire with a Valsalva manoeuvre during a telephone interview [8, 9]. However, further evidence is needed for online or app-based implementations. In future versions of the Q1.6 application, patients should be instructed to perform the Valsalva manoeuvre at home, preferably with the inclusion of instructional images or videos.

### Limitations

Although the Q1.6 application shows promise in recognising complications after inguinal hernia repair, this study does have several limitations. First, data obtained from the application were validated using retrospectively collected clinical data and additional telephone interviews. Ideally, prospective collection of clinical outcomes and standardised measurements with an equivalent follow-up duration would provide a more reliable assessment of safety and validity. Retrospective data retrieval may have led to data loss and patient dropout. In addition, the application suffers from missing data as patients had the option to skip questions and did not answer all questions preoperatively and postoperatively. During the pilot phase, patients were not obligated to use the application for a specific duration, leading to decreased compliance over time [4]. This potential bias in the cohort could impact the evaluation of long-term complications such as CPIP and recurrence. Moreover, standard care follow-up is insufficient to recognise these long-term complications. Furthermore, it was not always evident whether patients understood and answered questions accurately, and the frequency of errors in their responses remains uncertain. The questions themselves may be susceptible to misinterpretation or linguistic errors. Future research will address these limitations, focussing on repeated measurements to overcome incomplete and incorrect responses.

### Future aspects

The potential of a remote monitoring application for post-inguinal hernia repair is promising. However, further improvements and thorough cost, safety and validation investigations are necessary before integrating it into conventional medical care. Accurate content and appropriate question selection are vital in preventing errors and patient dropout. To enhance the application and address initial challenges, a comprehensive analysis of data, including linguistic examination and patient feedback regarding their experience with the application and interpretation of questions, is essential. The establishment of a core outcome set can be achieved by making it obligatory to answer key questions [29]. In a broader context, the assessment of costs for innovative healthcare modalities is crucial, with the aim being cost neutrality or reduction compared to standard care. The establishment of long-term financial plans is vital to ensure the success and cost-effectiveness of such initiatives.

## Conclusion

This study provides insights into the efficacy of the Q1.6 Inguinal Hernia application for identifying postoperative complications following inguinal hernia repair. The application demonstrates satisfactory measurement capabilities for several parameters and reveals data on functional outcomes and recovery that would have otherwise remained unknown. However, certain aspects require further improvement, such as addressing error-prone questions, enhancing long-term compliance, evaluating cost-effectiveness, and validating (pain) measurements through prospective control data. Future research on these topics is crucial to enhancing the Q1.6 application’s potential and making it a prominent tool in patient care and an alternative to conventional physical or telephone follow-up after inguinal hernia repair.

## Data Availability

The data that support the findings of this study are available on request of the corresponding author, L. van Hout.
